# Glucocorticoid attenuates acute lung injury through induction of type 2 macrophage

**DOI:** 10.1186/s12967-017-1284-7

**Published:** 2017-08-29

**Authors:** Guo-wei Tu, Yi Shi, Yi-jun Zheng, Min-jie Ju, Hong-yu He, Guo-guang Ma, Guang-wei Hao, Zhe Luo

**Affiliations:** 1 Department of Critical Care Medicine, Zhongshan Hospital, Fudan University, Shanghai, 200032 People’s Republic of China; 2 Biomedical Research Center, Zhongshan Hospital, Fudan University, Shanghai, People’s Republic of China

**Keywords:** Glucocorticoid, Methylprednisolone, Acute lung injury, Acute respiratory distress syndrome, Macrophage, Regulatory T cell

## Abstract

**Background:**

Acute lung injury (ALI) and acute respiratory distress syndrome (ARDS) are severe inflammatory lung diseases. Methylprednisolone (MP) is a common drug against inflammation in clinic. In this study, we aim to investigate the protective effect of MP on ALI and potential mechanisms.

**Methods:**

Male BABL/c mice were injected through tail vein using lipopolysaccharide (LPS, 5 mg/kg) with or without 5 mg/kg MP. Lung mechanics, tissue injury and inflammation were examined. Macrophage subsets in the lung were identified by flow cytometry. Macrophages were cultured from bone marrow of mice with or without MP. Then, we analyzed and isolated the subsets of macrophages. These isolated macrophages were then co-cultured with CD4^+^ T cells, and the percentage of regulatory T cells (Tregs) was examined. The expression of IL-10 and TGF-β in the supernatant was measured. The Tregs immunosuppression function was examined by T cell proliferation assay. To disclose the mechanism of the induction of Tregs by M2c, we blocked IL-10 or/and TGF-β using neutralizing antibody.

**Results:**

Respiratory physiologic function was significantly improved by MP treatment. Tissue injury and inflammation were ameliorated in the MP-treated group. After MP treatment, the number of M1 decreased and M2 increased in the lung. In in vitro experiment, MP promoted M2 polarization rather than M1. We then induced M1, M2a and M2c from bone marrow cells. M1 induced more Th17 while M2 induced more CD4^+^CD25^+^Fxop3^+^ Tregs. Compared with M2a, M2c induced more Tregs, and this effect could be blocked by anti-IL-10 and anti-TGF-β antibodies. However, M2a and M2c have no impact on Tregs immunosuppression function.

**Conclusion:**

In conclusion, MP ameliorated ALI by promoting M2 polarization. M2, especially M2c, induced Tregs without any influence on Tregs immunosuppression function.

## Background

Methylprednisolone (MP), as a potent long-lasting synthetic glucocorticoid, is widely used in clinical settings. MP inhibits inflammatory cascade in various pathophysiological conditions. Therefore, it is widely used to treat a variety of acute and chronic inflammatory diseases including acute lung inflammation, asthma, and rheumatoid arthritis [1]. A number of studies have been reported for the anti-inflammatory properties of MP [2–4]. For instance, MP induces anti-inflammatory cytokines expression such as IL-10. MP also suppresses pro-inflammatory cytokines expression such as IL-6, IL-8, TNF-α, and cell adhesion molecules, which are involved in the migration of leucocytes into the extravascular space [5].

Acute lung injury (ALI) and acute respiratory distress syndrome (ARDS) are severe inflammatory lung diseases due to lung injury from a variety of precipitants. Both of them might result from severe trauma or sepsis. Pathophysiologically, ALI is characterized by disruption of the alveolar-capillary interface, pro-inflammatory cytokines secretion, and pro-inflammatory cells infiltration. In addition, destruction of pneumocytes during ALI or ARDS causes subsequent fibrosis and hyalinization of the lung membrane [6]. Despite many research and therapeutic trials, no effective therapies are available for ALI in clinic. As so far, glucocorticoid treatment in the early phase of lung inflammation appears to resolve ALI and ARDS [7]. A larger clinical study is currently trying to determine the parameters for more effective glucocorticoid treatment and to develop appropriate guidelines (clinical trial NCT01731795).

Alveolar macrophages, as a prominent subset of innate immunocytes, play a key role in lung inflammation initiation, resolution and tissue repair [8]. Nowadays, different subsets of macrophages have been identified. The classically activated or M1 are pro-inflammatory macrophages, in response to bacterial components or IFN-γ and TNF-α. However, macrophage can also assume a variety of alternatively activated or M2 phenotypes [9]. This unique M2 subset owns different markers compared to M1, and contributes to inflammation resolution and tissue repair. Subsequently, additional stimuli that were previously considered to be macrophage deactivators are also shown to produce distinct macrophages activation phenotypes in vitro. The M2 macrophages are divided three new phenotypes, now frequently referred to as M2a/b/c. It has been reported that IL-4-mediated therapeutic macrophage reprogramming to M2 could accelerate resolution and lung repair following experimental ALI [10]. A recent study showed that adoptively transfer M2a or M2c into LPS-induced ALI mice significantly reduced lung inflammation and injury including a reduction of neutrophil influx into the lung and an augmentation of apoptosis [11]. Furthermore, in vivo depletion of CD206^+^ M2 macrophages exaggerates acute lung injury in mice model [12]. These results proved that M2 macrophages ameliorate inflammation and promote tissue repair in ALI.

Since M2 can be considered as a kind of regulatory cells in the innate immunity, it cannot ignore the effects in that involved regulatory T cells (Tregs) in ALI. Many effective therapeutic strategies for ALI are involved in expansion or induction Treg in vivo. For instance, transplantation of human umbilical cord mesenchymal stem cells enhanced the diminished levels of alveolar Treg and ameliorated ALI in mice [13]. Using an epigenetic regulation method, DNA methyltransferase inhibition augmented Treg number and function, and accelerated repair of experimental lung injury [14].

Despite glucocorticoid and M2 macrophages are beneficial for ALI therapy, the influence of glucocorticoid on macrophage differentiation is unclear, especially for M2. In the current study, we investigated the therapeutic effects of MP on lipopolysaccharide (LPS)-induced ALI, and whether MP regulates macrophages differentiation. At last, we revealed the mechanism that M2c induction Treg in vitro.

## Results

### MP improved lung function and ameliorated tissue injury

**Fig. 1:**
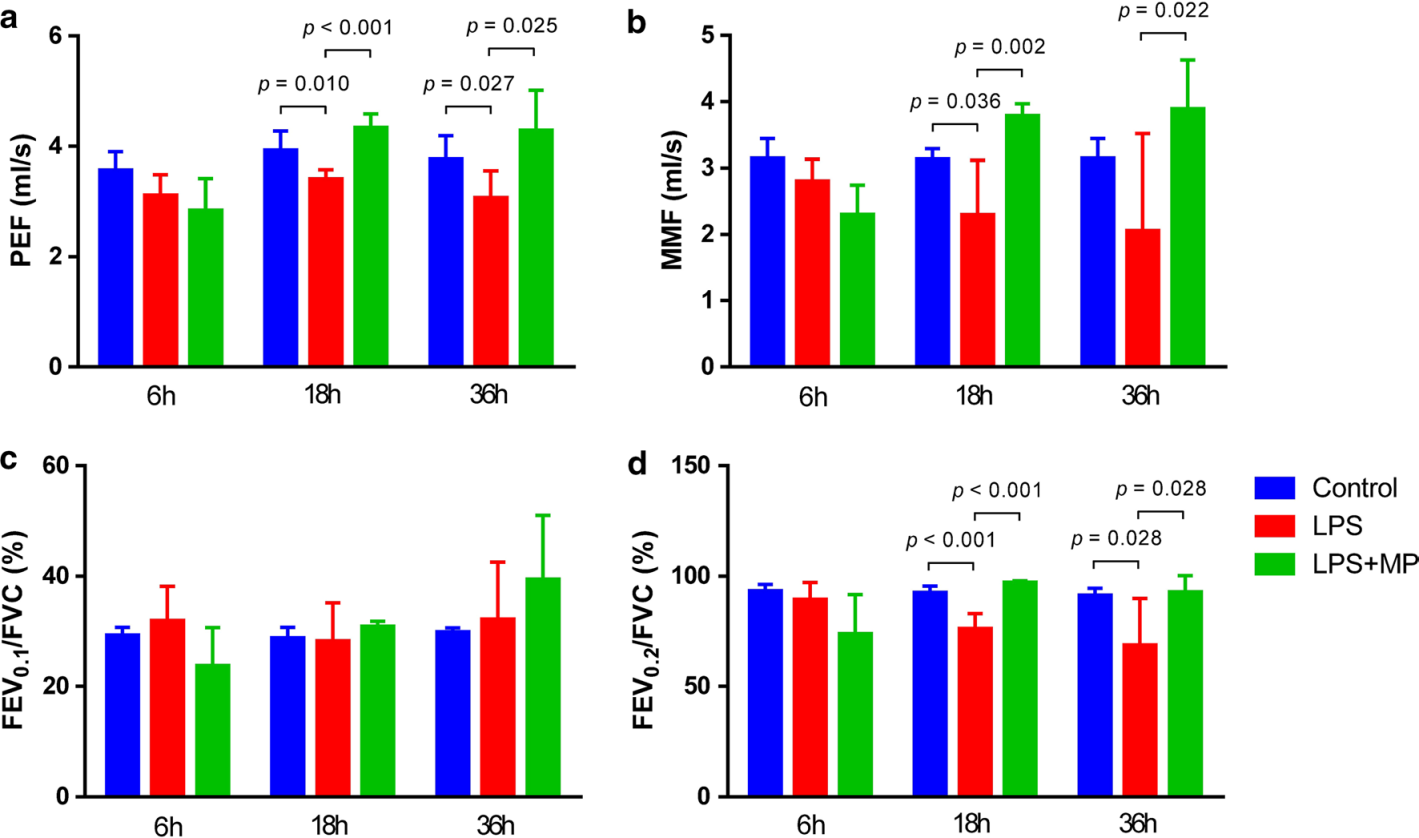
Lung function and ameliorated tissue injury. After the tracheostomy, we detected lung function at 6, 18 and 36 h in ALI model. Compared to the LPS group, **a** MP significantly increased the peak expiratory flow (PEF), **b** maximal mid-expiratory flow (MMF) and **d** forced expiratory volume 0.2 (FEV_0.2_)/forced vital capacity (FVC) at 18 and 36 h. **c** No significant difference was observed in forced expiratory volume 0.1 (FEV_0.1_)/forced vital capacity (FVC) among these groups. Data were shown as mean ± S.D.; n = 5 mice per group

**Fig. 2:**
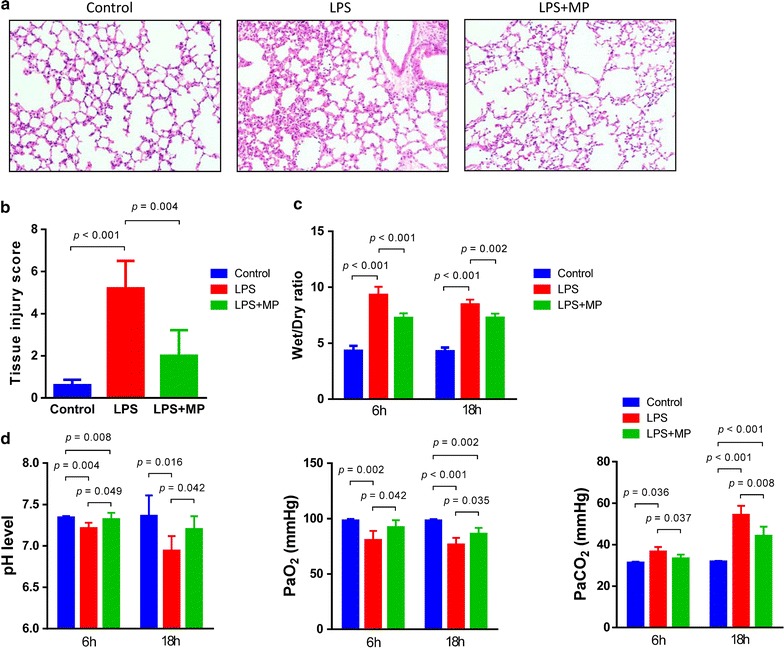
Tissue injury, lung weight/dry and arterial blood gas analysis. **a** At the 18 h after ALI, the lung was harvest and histologic damage was assessed in H&E-stained sections. **b** The semi-quantitative analysis of tissue injury indicated that MP treatment significantly attenuated tissue injury in the lung. **c** The severity of pulmonary edema was assessed by the wet to dry ratio (W/D ratio). MP treatment significantly reduced the W/D compared to that in the LPS group at 6 and 18 h. **d** Arterial blood gas analysis showed MP treatment significantly improved acid–base disturbance, increased PaO_2_ and reduced PaCO_2_. Data were shown as mean ± S.D.; n = 5 mice per group

We detected lung function at 6, 18 and 36 h in ALI model with or without MP treatment. Compared to the LPS group, MP significantly increased the peak expiratory flow (PEF) (Fig. 1a), maximal mid-expiratory flow (MMF) (Fig. 1b) and forced expiratory volume 0.2 (FEV_0.2_)/forced vital capacity (FVC) (Fig. 1d) at 18 and 36 h. At 18 h after ALI, H&E staining showed interstitial edema, interalveolar septal thickening and intra alveolar and interstitial patchy hemorrhage in the LPS group (Fig. 2a). After MP treatment, the tissue injury was significantly attenuated (Fig. 2b). As for pulmonary edema evaluation, lung weight and dry ratio (W/D) was employed. The mice received LPS injection showed higher W/D than that in the MP group (Fig. 2c). In addition, MP treatment significantly improved acid–base disturbance, increased PaO_2_ and reduced PaCO_2_ (Fig. 2d).

### MP ameliorated inflammation in vivo

**Fig. 3:**
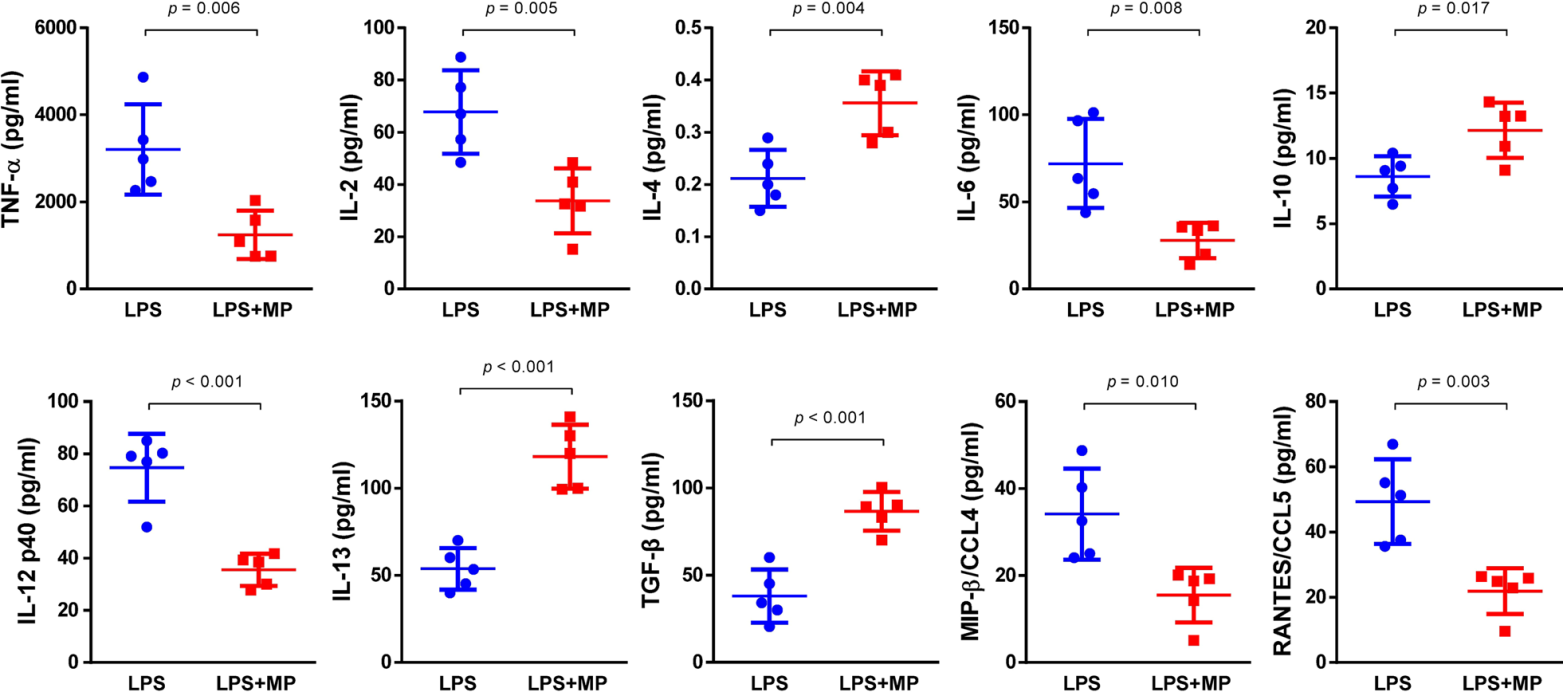
Cytokines and chemokines in (Bronchoalveolar lavage fluid) BAFL. The BALF supernatant was collected after centrifugation at 18 h after ALI. The expression of TNF-α, IL-2, IL-4, IL-6, IL-10, IL-12p40, IL-13, TGF-β, CCL4 and CCL5 in BAFL was measured by ELISA. The expression of TNF-α, IL-2, IL-6, IL-12p40, CCL4 and CCL5 decreased in the MP-treated group. However, MP treatment increased the level of IL-4, IL-10, IL-13 and TGF-β. Data were shown as mean ± S.D.; n = 5 mice per group. *ELISA* enzyme-linked immuno sorbent assay

To determine the inflammation level, we detected typical pro-inflammatory cytokines and chemokines in BAFL at 18 h after ALI. The expression of TNF-α, IL-2, IL-6, IL-12p40, CCL4 and CCL5 decreased in the MP-treated group. However, MP treatment increased the level of IL-4, IL-10, IL-13 and TGF-β (Fig. 3). These results suggested that MP inhibited the pro-inflammatory response, and reconstituted inflammatory microenvironment.

### MP regulated the ratio of macrophage subsets in vivo

**Fig. 4:**
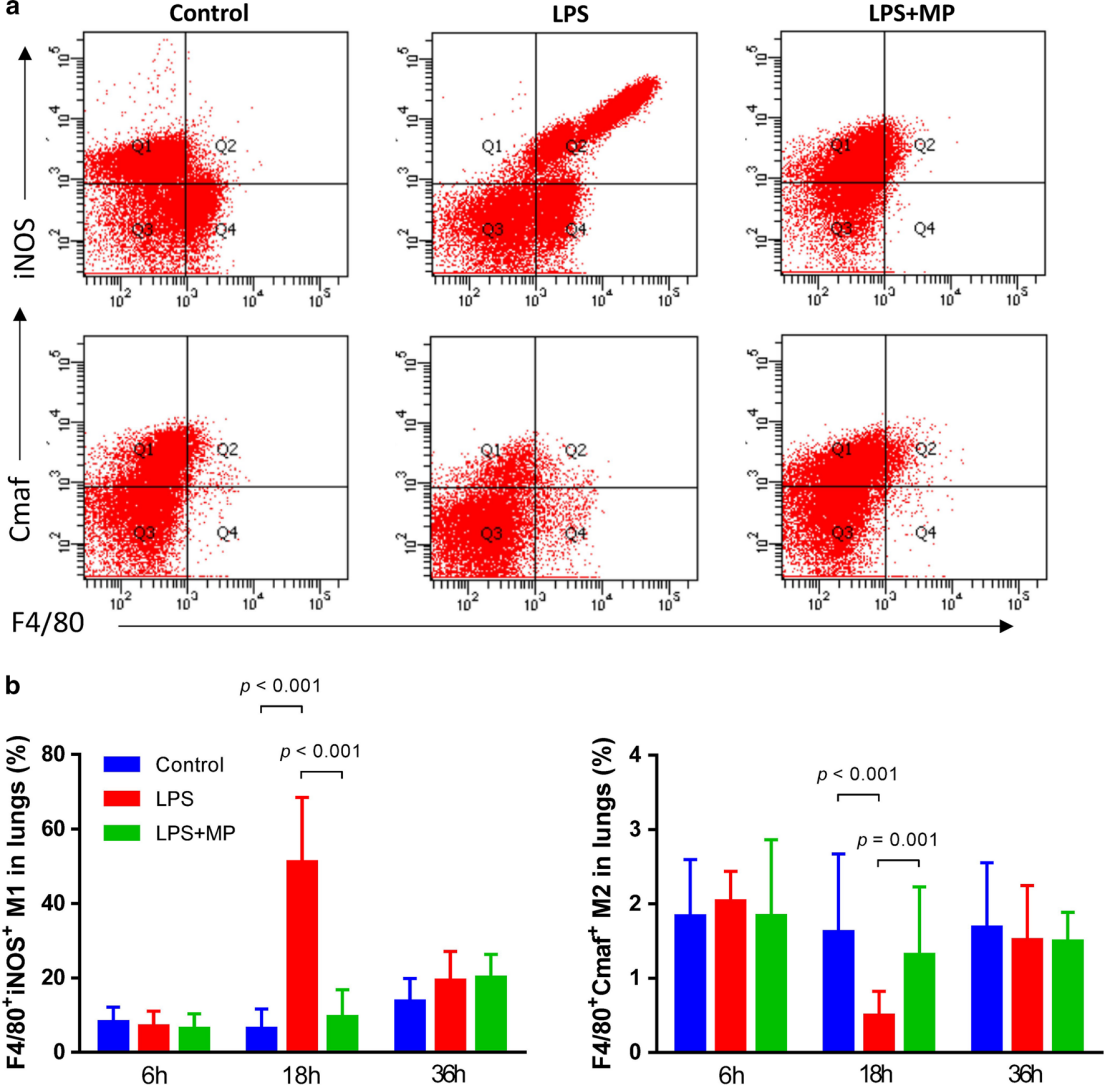
Macrophage subsets in vivo. After separating single cell in lung, flow cytometry was performed. **a** The percentage of F4/80^+^iNOS^+^ M1 and F4/80^+^Cmaf^+^ M2 were detected by flow cytometry of in the lung. **b** Semi-quantitative analysis showed F4/80^+^Cmaf^+^ M2 was decreased in the LPS group. MP treatment reduced M1 and increased M2 in the lung. No difference was observed between the control and MP treatment groups at 6 and 36 h. Data were shown as mean ± S.D.; n = 5 mice per group

Next, we investigated the ratio of M1/M2 in the lung by flow cytometry (Fig. 4a). At 18 h after LPS injection, F4/80^+^iNOS^+^ M1 was significantly increased. Cmaf is an appropriate marker of M2 [15], so we gated Cmaf from F4/80^+^ cells. In the LPS group, F4/80^+^Cmaf^+^ M2 was decreased. However, MP treatment reduced M1 and increased M2 in the lung. No difference was observed between the control and MP treatment groups at 6 and 36 h (Fig. 4b), suggesting MP administration restored the balance of macrophage subsets at the middle stage after ALI.

### MP promoted M2 differentiation in vitro

**Fig. 5:**
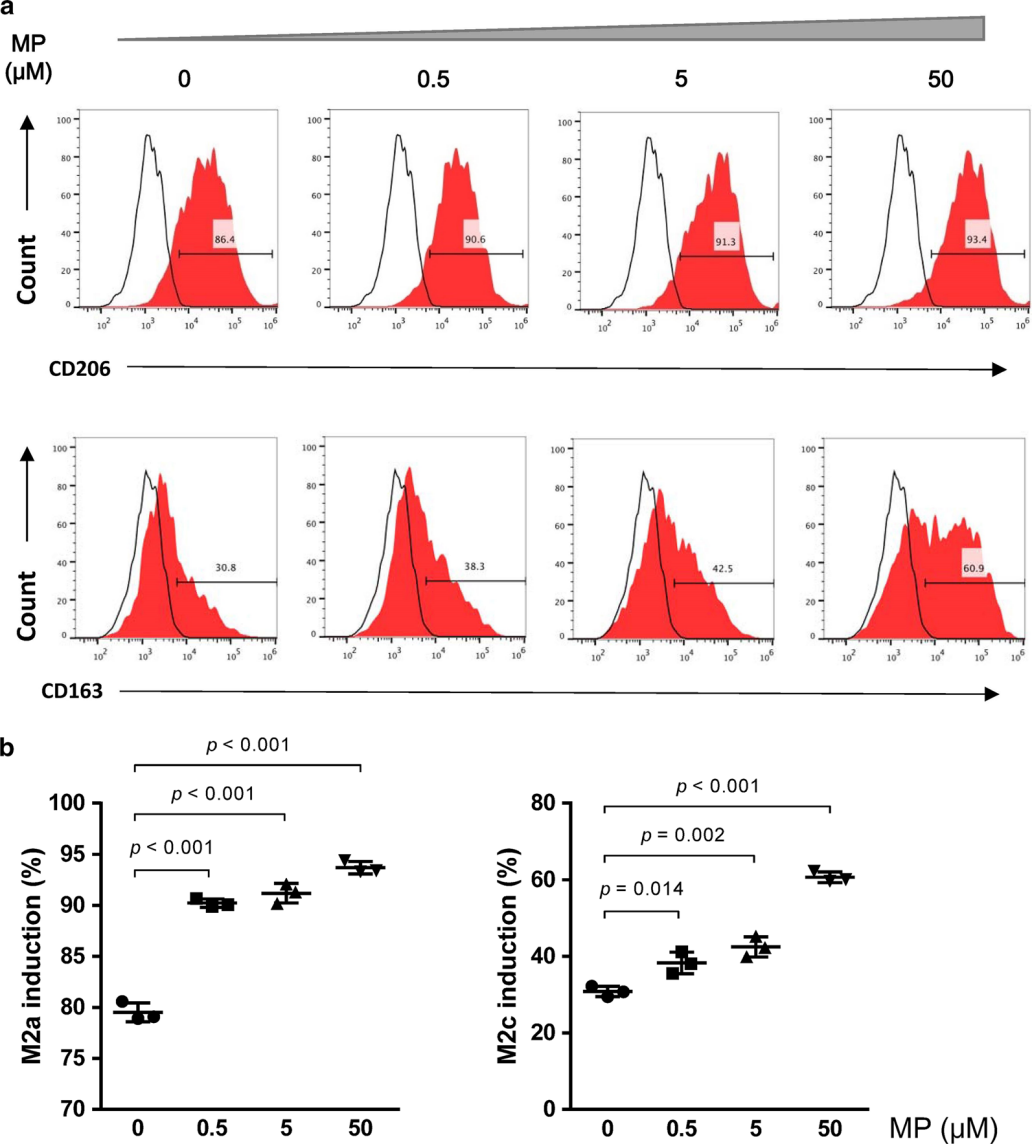
M2a and M2c differentiation in vitro. **a** Different dose of MP was added during M2a and M2c induction culture. The flow cytometry showed the percentage of CD206^+^ M2a and CD163^+^ M2c. **b** MP increased the CD206^+^ M2a and CD163^+^ M2c percentage in a dose-dependent way

Since MP increased the M2 percentage in vivo, we then further investigated whether MP directly regulates macrophage differentiation in vitro. It has been demonstrated that adoptively transfer M2a or M2c into LPS-induced ALI mice reduced ALI [11]. We, therefore, detected the M2a and M2c percentage with different dose of MP (Fig. 5a). In the presence of IL-4 and IL-13, MP increased the CD206^+^ M2a percentage in a dose-dependent way (Fig. 5b). After stimulation by IL-10 and TGF-β, the percentage of CD163^+^ M2c was also increased in the MP-treated group. The trend presented in a dose-dependent way (Fig. 5b). This result indicated that MP promoted M2a and M2c differentiation.

### M2c induced more Tregs than M2a

**Fig. 6:**
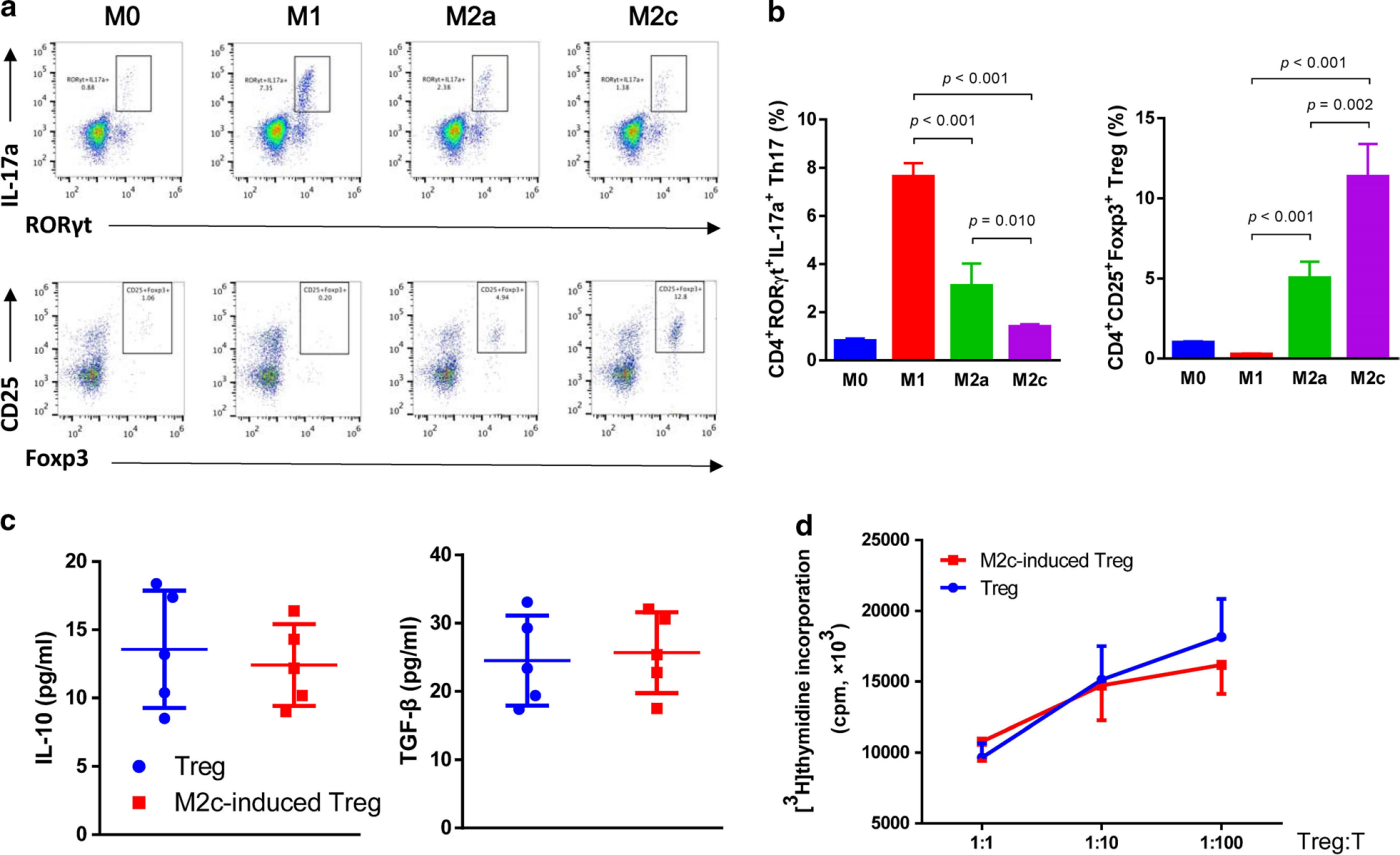
Influence on Tregs differentiation and function by macrophages. **a** Induced-M2a and M2c were isolated by flow cytometry and co-cultured with CD4^+^ naïve T cells, as well as M0 and M1, under Tregs induction environment. CD4^+^CD25^+^Foxp3^+^ Tregs and CD4^+^IL-17a^+^RORγt^+^ Th17 were detected. **b** Semi-quantitative analysis of Th17 and Tregs percentage showed M1 induced much more percentage of Th17. Compared to M0 and M1, both M2a and M2c significantly induced Tregs and inhibited Th17. Intriguingly, M2c induced more Tregs than M2a. **c** These M2c-induced Tregs were isolated. Compared to the normal Tregs, the expression of IL-10 and TGF-β in supernatant has no difference in M2c-induced Tregs. **d** Mix lymphocyte reaction was performed to compare the immunosuppression function between the two groups of Tregs. The immune suppressive function between normal Tregs and M2c-induced Tregs showed no significant difference

Tregs play an important role in inflammation resolution in ALI [16, 17]. We assumed M2 induce Tregs and ameliorated inflammation. Therefore, these induced-M2a and M2c were isolated by flow cytometry and co-cultured with CD4^+^ naïve T cells under Tregs induction environment. We detected CD4^+^CD25^+^Foxp3^+^ Tregs and CD4^+^IL-17a^+^RORγt^+^ Th17 (Fig. 6a). As expected, the pro-inflammatory M1 induced much more percentage of Th17. Compared to M0 and M1, both M2a and M2c significantly induced Tregs and inhibited Th17. Intriguingly, M2c induced more Tregs than M2a (Fig. 6b). To clarify the impact on Tregs immunosuppression function by M2, we further isolated Tregs that were induced by M2c. Compared to the normal Tregs, the expression of IL-10 and TGF-β in supernatant has no difference in M2c-induced Tregs (Fig. 6c). At last, mix lymphocyte reaction was performed to compare the immunosuppression function between the two groups of Tregs. The result further confirmed that M2c did not influence on Tregs immunosuppression function although increased their number (Fig. 6d).

### M2c induced Tregs through IL-10 and TGF-β secretion

**Fig. 7:**
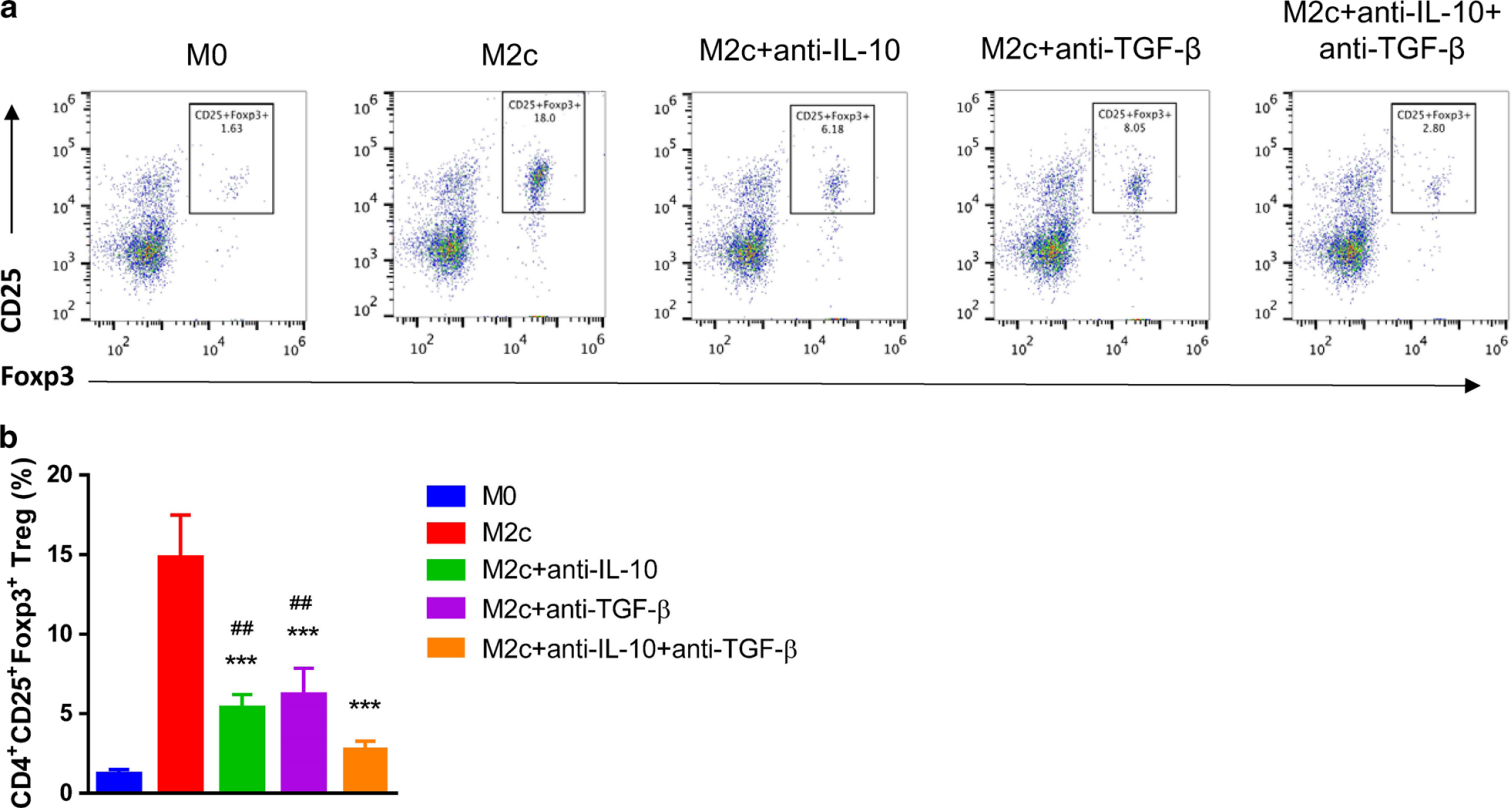
M2c induced Tregs through IL-10 and TGF-β secretion. **a** Anti-IL-10 and/or anti-TGF-β antibodies were added in the co-culture system. **b** Either anti-IL-10 or anti-TGF-β antibody significantly reversed Tregs induction by MP. In the mixed antibodies group, the number of Tregs is the lowest

To elucidate the mechanism that M2c induces Tregs, we added anti-IL-10 and/or anti-TGF-β antibodies in the co-culture system (Fig. 7a). Either anti-IL-10 or anti-TGF-β antibody significantly reversed Tregs induction. In the mixed antibodies group, the number of Tregs is the lowest (Fig. 7b). The result indicated that IL-10 or TGF-β pathway is the key mechanism that involved in the induction of Tregs by M2c.

## Discussion

In this study, we found that MP significantly improved respiratory function after LPS insult. MP also prompted macrophage toward M2 polarization. Furthermore, M2c was the dominant subset that induced by MP treatment. In addition, M2c could induce Treg in vitro in terms of increased number but without any influence on immune suppression function.

Glucocorticoid treatment is an effective therapy for ALI and ARDS. In our clinical study, methylprednisolone was used routinely for renal transplant recipients with severe ALI or ARDS. All immunosuppressants were discontinued at admission to ICU and methylprednisolone (1 mg/kg every 12 h) was initiated followed by gradual tapering [18, 19]. We found over-reactive inflammation cascade in these ALI/ARDS patients was inhibited by MP treatment. We also found that moderate-dose MP played an important role as salvage therapy for these patients [20].

Since MP therapy is effective in clinical settings, we further investigated whether MP regulated innate immunity in ALI/ARDS. In the present study, we found the significant change of M1/M2 ratio in ALI. In detail, M1 increased and M2 decreased during the pathologic process, which suggested a break of balance between M1 and M2. Interestingly, at the very early stage, the ratio between M1 and M2 did not have any remarkable change, however, the M1/M2 ratio increased at 18 h post-ALI. The result was in keeping with pulmonary function and inflammatory cytokines expression profile. After MP treatment, the percentage of M2 increased accompanied by decreased M1, suggested MP helped to reconstituted balance between M1 and M2.

**Fig. 8:**
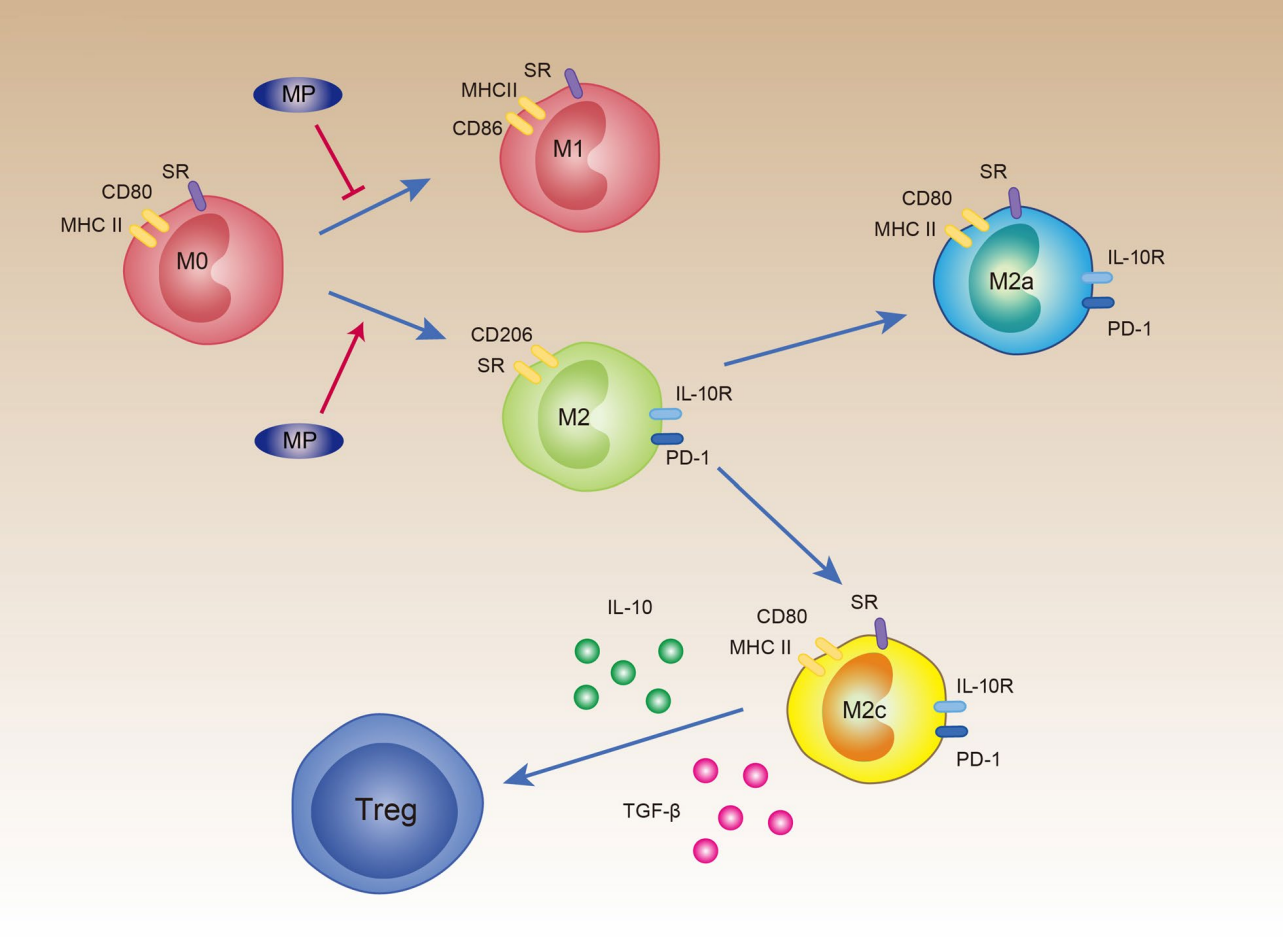
Schematic picture of the mechanism. In ALI model, MP treatment restores the balance between M1 and M2. MP promotes M2 differentiation in terms of M2a and M2c. Compared to M2a, MP-induced M2c induces more Tregs. Regulation innate and adaptive immune response by MP might be a mechanism of ameliorating ALI

Recent studies have focused on the diverse role of macrophages and, in particular, their ability to regulate inflammation and tissue repair in various disease including autoimmunity, atherosclerosis, microbial infection, parasitic infection, and cancer [10, 21]. Although macrophage could be divided into M1 and M2, M2 can further be classified into three subsets based on their in vitro response to stimuli: M2a incubated with IL-4 and IL-13, M2b with immune complexes, and M2c with IL-10 and TGF-β. A study by Nelson et al. indicated that experimental polarization of naive alveolar macrophages to M2a resulted in more efficient killing of *P. murina* compared with untreated alveolar macrophages, which was further enhanced by the addition of IL-33 [22]. Recently, Tang et al. investigated different M2 subsets therapeutic effects on experimental ALI. M2a and M2c were adoptively transferred into LPS-induced ALI mice model. Both subsets significantly reduced lung inflammation and injury including a reduction of neutrophil influx into the lung and an augmentation of apoptosis [11]. Interestingly, M2c macrophages more effectively suppressed indices of lung injury than M2a macrophages. M2c macrophages were also more effective than M2a in the reduction of lung fibrosis. M2c but not M2a macrophages increased IL-10 level in lung tissues of the recipient ALI mice. After blocking IL-10, these superior effects of M2c over M2a were abolished [11]. In our study, we found that M2c induced significant more Tregs than M2a, which might partially explain that M2c was more effective in amelioration tissue injury in ALI in Tang’s study. Treg differentiation needs the third signal by cytokines in the microenvironment. TGF-β is a well-known cytokine that promotes naïve CD4 T cell to differentiate into Treg [23]. Although IL-10 is the dominant secreting cytokine by Treg, it is also able to promote Treg differentiation. For instance, IL-10 secreted by HO-1-expressing DCs at a high level may enhance type 1 Treg (Tr1) differentiation, which has a critical role in controlling peripheral tolerance [24]. CD11c^+^CD11b^+^CD8^−^ DCs produce elevated levels of IL-10 after *P. yoelii* infection. Conditional knockout of *Il*-*10* in DCs interfered with the induction of Tregs [25]. Therefore, by blocking IL-10 and TGF-β, we demonstrated that induction of Treg by M2c was through IL-10 and TGF-β pathway (Fig. 8). These induced-Tregs participate in inflammation resolution and tissue repair after ALI [26, 27].

In conclusion, MP treatment ameliorates acute lung injury and inflammation in LPS-induced ALI model. MP also regulates and reconstitutes the balance between M1 and M2. In mechanism, these induced M2a and M2c by MP secret IL-10 and TGF-β, which could then induce Treg. In addition, M2c induced more Treg than M2a.

## Methods

### Animals

All animal use procedures were approved by the Committee on the Ethics of Animal Experiments of Zhongshan Hospital, Fudan University. This investigation was carried out in strict accordance with the recommendations in the Guide for the Care and Use of Laboratory Animals of the National Institutes of Health. All surgery was performed under sodium pentobarbital anesthesia, and all efforts were made to minimize suffering. Six weeks old specific pathogen-free male BALB/c mice (16–20 g) were maintained under specific pathogen-free conditions in the facilities of the animal center. The mice were kept in a temperature controlled room with 12 h dark/light cycles, and allowed food and water ad libitum. Animals underwent an acclimatization period of at least 14 days before use in our study.

### ALI model

A total of 35 BALB/c mice were randomly divided into three main groups, control group (n = 5), LPS (Sigma, St. Louis, MO, USA) group (n = 15), MP (Pfizer, New York, NY, USA) treatment group (n = 15). For LPS and MP groups, the mice were subdivided into six subgroups (n = 5). Mice were sacrificed at 6, 18 and 36 h. ALI was induced by LPS via intravenous injection as previously described [28–32]. The mice in control group were administrated the sterile saline instead. Ten minutes after LPS injection, MP was given by intraperitoneal injection at 5 mg/kg in a single dose.

### Measurement of lung mechanics

Each mouse was weighed, anesthetized by an intraperitoneal injection of pentobarbital sodium (40 mg/kg). Tracheostomy was performed and the trachea was cannulated with an 18-gauge intravenous catheter. After the tracheostomy, mice were placed in a body plethysmograph and connected to a computer-controlled ventilator. Lung mechanics were analyzed by the system of AniRes2005 (Bestlab High-Tech Co., Ltd, Beijing, China).

### Lung wet/dry weight ratio

The severity of pulmonary edema was assessed by the wet to dry ratio (W/D ratio). The right lower lungs weighed and then dehydrated at 60 °C for 72 h in an oven.

### Tissue injury score and arterial blood gas analysis

The left lower lung from each mouse was fixed in 10% formalin, embedded in paraffin, cut into 5 mm sections, stained with H&E. Lung injury score was measured by a blinded pathologist with a 0–4 point scale according to combined assessments of inflammatory cell infiltration in the airspace or vessel wall, alveolar congestion, hemorrhage, alveolar wall thickness and hyaline membrane formation. A score of 0 represented no damage; l represented mild damage; 2 represented moderate damage; 3 represented severe damage and 4 represented very severe histological changes. We collected blood from the abdominal aorta and performed arterial blood gas analysis.

### Cytokines expression in bronchoalveolar lavage fluid

Bronchoalveolar lavage fluid (BALF) was collected at 18 h after LPS injection by cannulating the upper part of the trachea, by lavage three times with 1.0 ml PBS (pH 7.2). The fluid recovery rate was more than 90%. Lavaged sample from each mouse was kept on ice. BALF was centrifuged at 700*g* for 5 min at 4 °C. The BALF supernatant was collected after centrifugation (for 4 min at 4000 rpm) and stored at −80 °C before cytokine assay. TNF-α, IL-2, IL-4, IL-6, IL-10, IL-12 (p40), IL-13, TGF-β, CCL4 and CCL5 were measured by ELISA (R&D Systems, Minneapolis, MN, USA) as previously described [33].

### Flow cytometry

The single cell suspension of lung was prepared as previously described [34]. Thereafter all single cells were resuspended in staining buffer (BD Bioscience, San Diego, CA, USA). The following monoclonal antibodies were added into the single-cell suspension according to manufacturers’ protocols: F4/80, CD163, CD206, Cmaf (eBioscience, San Jose, CA, USA). For Th17 and Treg detection, cells were stained with surface markers, fixed and permeabilized with fixation buffer [Dulbecco’s phosphate-buffered saline (pH 7.4) with 4% w/v paraformaldehyde (0.22 µm pore-filtered)]/permeabilization buffer [PBS, 1% fetal calf serum, 0.1% sodium azide, 0.1% saponin (0.2 µm pore-filtered; pH7.4-7.6); eBioscience] and then stained with RORγt and Foxp3 antibodies (eBioscience).

### Cytokines in cell culture supernatant

The supernatant was collected and stored at −80 °C until further analysis. The level of IL-10 and TGF-β was measured using Mouse IL-10 and TGF-β ELISA Kit (Abcam, Cambridge, UK) in accordance with the manufacturer’s protocol respectively as previously described [35].

### Macrophage culture and differentiation

BALB/c mice splenocytes were harvested and triturated with sterile syringes, and the resulting cell suspension was filtered through 40 mm nylon mesh and was incubated at 37 °C for 40 min, and then the culture supernatant that contained floating cells (T cells, natural killer cells, and dendritic cells) was discarded. The adherent spleen-derived macrophages were rinsed three times in RPMI-1640 medium and cultured for 48 h with the normal medium to be M0 (resting macrophages), with IL-4/IL-13 (10 ng/ml each) to become M2a and with IL-10/TGF-β (10 ng/ml each) to become M2c macrophages. IL-4, IL-13, IL-10, and TGF-β were purchased from Invitrogen (Mount Waverly, Australia).

### Mixed lymphocyte reaction

Mononuclear cells (MNCs) of spleens from mice were obtained by Ficoll density gradient centrifugation. CD4^+^ T cells were magnetically purified from MNCs according to the manufacturer’s recommendations (Miltenyi Biotec, Auburn, CA, USA). The purity of sorted cells in this study was consistently more than 95%. Tregs with or without M2c induction were lethally irradiated (30 Gy). The Tregs were then cultured in graded doses with CD4^+^ T cells (3 × 10^5^ cells/well) in RPMI 1640 medium for 5 days. [^3^H]Thymidine (1 μCi/well, Shanghai Institute of Applied Physics, Chinese Academy of Sciences, China) was added 18 h before the end of the culture period. The cells were then harvested onto glass fiber mats for the measurement of [^3^H]thymidine incorporation.

### Statistical analysis

Results are expressed as the mean ± standard deviation (S.D.). Statistical analysis was performed using the Student’s *t* test (between two groups) and one-way ANOVA (among three or more groups) by SPSS 19.0 software (SPSS, Inc., Armonk, NY, USA). The Scheffe test was used for post hoc analysis. P < 0.05 was recognized as statistically significant.

## Abbreviations

ALI: acute lung injury
ARDS: acute respiratory distress syndrome
MP: methylprednisolone
Tregs: regulatory T cells
LPS: lipopolysaccharide
PEF: peak expiratory flow
FEV_0.2_: forced expiratory volume 0.2
FVC: forced vital capacity
W/D: weight and dry ratio
